# COVID-19 patients exhibit unique transcriptional signatures indicative of disease severity

**DOI:** 10.3389/fimmu.2022.989556

**Published:** 2022-09-15

**Authors:** Andrea R. Daamen, Prathyusha Bachali, Catherine A. Bonham, Lindsay Somerville, Jeffrey M. Sturek, Amrie C. Grammer, Alexandra Kadl, Peter E. Lipsky

**Affiliations:** ^1^ AMPEL BioSolutions LLC, Charlottesville, VA, United States; ^2^ Department of Medicine, Division of Pulmonary and Critical Care Medicine, Charlottesville, VA, United States; ^3^ Beirne B. Carter Center for Immunology Research, University of Virginia, Charlottesville, VA, United States; ^4^ Department of Pharmacology, University of Virginia, Charlottesville, VA, United States

**Keywords:** COVID-19, severity, classification, transcriptomics, bioinformatics

## Abstract

COVID-19 manifests a spectrum of respiratory symptoms, with the more severe often requiring hospitalization. To identify markers for disease progression, we analyzed longitudinal gene expression data from patients with confirmed SARS-CoV-2 infection admitted to the intensive care unit (ICU) for acute hypoxic respiratory failure (AHRF) as well as other ICU patients with or without AHRF and correlated results of gene set enrichment analysis with clinical features. The results were then compared with a second dataset of COVID-19 patients separated by disease stage and severity. Transcriptomic analysis revealed that enrichment of plasma cells (PCs) was characteristic of all COVID-19 patients whereas enrichment of interferon (IFN) and neutrophil gene signatures was specific to patients requiring hospitalization. Furthermore, gene expression results were used to divide AHRF COVID-19 patients into 2 groups with differences in immune profiles and clinical features indicative of severe disease. Thus, transcriptomic analysis reveals gene signatures unique to COVID-19 patients and provides opportunities for identification of the most at-risk individuals.

## Introduction

Coronavirus Disease 2019 (COVID-19) is caused by the RNA virus severe acute respiratory syndrome coronavirus 2 (SARS-CoV-2), which mediates respiratory infections and lung pathology of varying severity (1–3). Infected individuals may be asymptomatic or present with a range of mild symptoms that can be treated at home to severe manifestations requiring hospitalization (4–8). Among hospitalized patients, there remain differences in the degree of respiratory distress and the need for mechanical ventilation. This heterogeneity in COVID-19 patients necessitates the ability to identify risk factors for severe disease and the development of acute hypoxic respiratory failure (AHRF). Currently accepted risk factors for worse clinical prognosis of COVID-19 patients include age, male gender, obesity, pre-existing diabetes, viral load, and pre-existing respiratory conditions or immunodeficiencies (9–11). However, these factors are not predictive of disease severity in all cases and young individuals in good health may still succumb to AHRF.

In addition to demographic statistics, the immune response of COVID-19 patients has been linked to disease severity and presents an opportunity for utilizing immune profiles to predict patient outcomes. To date, several groups have used a combination of flow cytometry, transcriptome data, cytokine levels, and clinical data to categorize COVID-19 patients and to associate particular immune profiles with disease severity (12–30). Immune cells and inflammatory molecules have been implicated in COVID-19 progression, including type I interferon (IFN) (22, 25, 27, 29), innate immune cells (12–14, 16, 18, 28), antibodies (31, 32), autoantibodies (33, 34), and pro-inflammatory cytokines (14, 17, 21, 23, 26, 35). However, these studies often describe conflicting associations of immune profiles with disease, emphasizing the need to better understand the heterogeneity in responses to SARS-CoV-2 infection. Furthermore, little work has focused on the classification of severe COVID-19 patients and the immune profiles associated with greater risk of death as a way to tailor treatment plans to each individual.

We previously utilized publicly available gene expression data to characterize the trajectory of the host immune response to SARS-CoV-2 in the blood, postmortem lung tissue, and bronchoalveolar lavage fluid (BALF) of COVID-19 patients (36). We have now employed a similar bioinformatic approach, combining gene expression and clinical feature data, to classify severe COVID-19 patients with AHRF upon admission to the intensive care unit (ICU) and to differentiate mild from severe patients at different timepoints after disease onset based on their immune profiles. In addition, we used longitudinal gene expression analysis from the same ICU patients to assess the stability of immune signatures over time. As a result, we identified two groups of AHRF COVID-19 patients with distinct enrichment of gene signatures of innate and adaptive immune cells and inflammatory pathways linked with differences in clinical outcomes. This classification of severe COVID-19 patients based on differences in immune profiles offers opportunities for identification of individuals with heightened disease severity and enables a targeted therapeutic approach to employ the most effective therapy for each individual COVID-19 patient.

## Materials and methods

### Patient population

We included patients who consented to donate blood to the University of Virginia (UVA) Intensive Care Unit (ICU) Biorepository. We then selected patients with confirmed COVID-19 respiratory failure, viral, non-COVID-19 respiratory failure, and patients with non-viral causes of respiratory failure (presumed bacterial infections) in the UVA ICU Biorepository to serve as a comparison cohort. Control patients were patients admitted on mechanical ventilation without respiratory failure (usually intubated for airway protection). We followed the Strengthening the Reporting of Observational Studies in Epidemiology (STROBE) guidelines (37) and our study complied with all principles outlined in the Declaration of Helsinki. All study protocols were approved by the UVA Institutional Review Board for Health Sciences Research (Protocol #21101). Respiratory failure was defined as patients with acute respiratory distress syndrome (ARDS) using Berlin criteria (38) who were on mechanical ventilation in the ICU. COVID-19 diagnoses were confirmed by RealTime SARS-CoV-2 assay performed on the m2000 system (Abbott Molecular Inc.; Des Plaines, IL).

### Serum cytokine and chemokine analysis

Serum cytokine and chemokine levels were measured by Merck Millipore MILLIPLEX Human Cytokine/Chemokine/Growth Factor Panel A (HCYTA-60K) and Panel II (HCYP2MAG) assays.

### Sample collection

Blood was collected into PAXgene® Blood RNA tubes upon admission to the ICU, and after 24 and 72 hours. RNA was prepared and sequenced by Genewiz (Azenta Life Sciences). RNA was isolated using a Qiagen total RNA isolation kit, followed by RNA-seq library preparation using standard Illumina protocols with rRNA and globin depletion. RNA was sequenced on an Illumina HiSeq 4000. 

### RNA-seq data analysis

Three independent whole blood datasets were analyzed. In the first dataset of patients from the UVA ICU Biorepository, RNA-seq data was obtained from 13 COVID-19 AHRF patients, 8 viral AHRF, 5 non-viral AHRF, and 5 control ICU patients. COVID-19 patients were subdivided into 2 groups (COVID Group 1 and COVID Group 2) based on separation by PCA of the top 500 variable genes. Additional publicly available RNA-seq datasets (GSE161731; GSE172114), were obtained and analyzed as confirmation studies. Additional dataset details can be found in **Supplementary Table 1**.

For RNA-seq analysis, the quality of raw FASTQ reads was analyzed using FASTQC (39) to identify the poor-quality reads and the adaptor contamination. Adaptors and low-quality sequencing reads were trimmed using Trimmomatic (40) and reads before 14bp were discarded. The clean raw sequencing reads were aligned to human reference genome (Gencode hg38) using STAR(v2) (41). The SAM files were converted into BAM files using sambamba (42). The aligned BAM files were fed to read summarization program featureCounts (43), to assign the sequencing reads to genomic features. The differential gene expression between COVID-19 and normal patients was carried out using the R/Bioconductor package DESeq2. The raw counts were normalized within the DESeq2 analysis pipeline using the median of ratios method and genes with low expression were filtered using HTSFilter (44). The p-values and adjusted p-values (FDR) were calculated for each gene. Genes with FDR < 0.05 were determined as significant differentially expressed genes. PCA plots of log_2_ transformed gene expression values were generated using the plotPCA function from the DESeq2 package.

### Gene set variation analysis (GSVA)

The R/Bioconductor package GSVA (45) (v1.25.0) was used as a non-parametric, unsupervised method to estimate the variation in enrichment of pre-defined gene sets in RNA-seq dataset samples as previously described (36) (www.bioconductor.org/packages/release/bioc/html/GSVA.html). In brief, a matrix of log_2_ transformed gene expression values for each sample and pre-defined gene sets were used as inputs for the GSVA algorithm. Then enrichment scores (GSVA scores) for each gene set were calculated using a Kolmogorov Smirnoff (KS)-like random walk statistic. GSVA scores for each patient and control were calculated and normalized to scores between -1 (no enrichment) and +1 (enriched). Significance of gene set enrichment between cohorts was calculated using a Welch’s t-test and p-value < 0.05 was considered significant.

Input gene sets used for GSVA analysis were previously used for the analysis of COVID-19 patient datasets (36) and can be found in **Supplementary Table 4**.

### Linear regression analysis

Multivariable linear regression analysis was performed with MaAsLin 2 (46), a Bioconductor R package that helps to determine the association between gene expression and complex metadata features. MaAsLin 2 is a statistical method that relies on general linear regression models which can test for the association between various functional and cell specific modules versus individual discrete and categorical clinical variables. Computed GSVA scores and patient metadata were used as input for the MaAsLin 2 function in R with normalization method and transformation method applied “NONE”, analysis method “LM”, and correction method “BH”. The significant associations with clinical variables were visualized using scatterplots and box plots.

Additional linear regression analyses for individual patient cohorts and between PC GSVA scores and log_2_ expression of Ig heavy chain transcripts were performed in GraphPad Prism (v 9.1.0; www.graphpad.com). For each analysis, the r^2^ value indicating the Goodness of Fit and the p-value testing the significance of the slope are displayed.

### Statistical analysis

Patient demographic data from COVID Group 1 and Group 2 were compared using an unpaired t-test with Welch’s correction for continuous variables. Pearson’s chi-squared tests were used to analyze count data between groups. Cytokine data for COVID Group 1, COVID Group 2, and control ICU patients was compared using Brown-Forsythe and Welch ANOVA tests with Dunnett T3 test for multiple comparisons. Significant differences in gene set enrichment between COVID-19 patients and non-COVID-19 patient cohorts were calculated using Brown-Forsythe and Welch ANOVA tests with Dunnett T3 test for multiple comparisons.

All statistical analyses were performed in GraphPad Prism (v 9.1.0; www.graphpad.com) and a two-tailed p-value of 0.05 was used for statistical significance.

### Data and materials availability

The RNA-seq data generated in the current study are publicly available through the National Center for Biotechnology Information (NCBI) under SRA project number PRJNA777938. The confirmation study datasets are publicly available under accessions GSE161731 and GSE172114.

## Results

### Transcriptomic analysis differentiates critically ill COVID-19 patients from other patients with AHRF and control patients in the ICU

**Figure 1:**
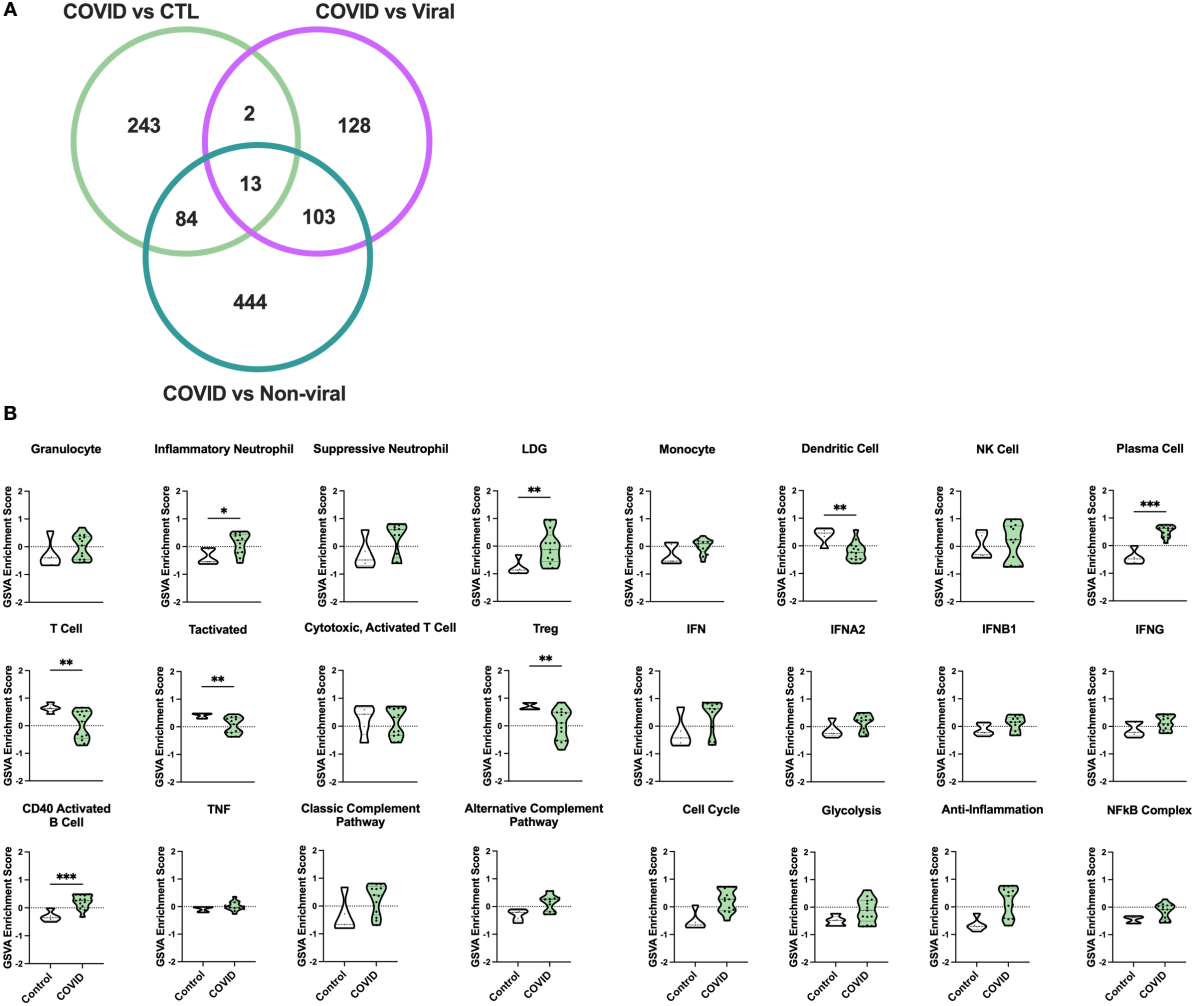
Gene signature analysis differentiates COVID-19 AHRF patients and control ICU patients. **(A)** Venn diagram of differentially expressed genes between COVID-19 patients and other ICU cohorts. **(B)** Individual sample gene expression from COVID-19 and control ICU patients was analyzed by GSVA for enrichment of immune cell and pathway gene signatures. Enrichment scores are shown as violin plots. *p < 0.05, **p < 0.01, ***p < 0.001.

To identify differences in immune pathology among severe COVID-19 patients, we analyzed whole blood transcriptomes from 13 individuals with COVID-19-induced AHRF, 6 with other viral and 7 with non-viral-induced AHRF, and 5 controls, who were patients admitted to the ICU on mechanical ventilation but without evidence of AHRF (**Supplementary Table 1**, **Supplementary Table 2**). Out of 18,130 total transcripts, we found 344 differentially expressed genes (DEGs) between control and COVID-19 ICU patients, 248 DEGs between viral AHRF and COVID-19 patients, and 650 DEGs between non-viral AHRF and COVID-19 patients (**Supplementary Table 3** and **Figure 1A**). Notably, expression of none of the genes previously associated with severe disease or increased mortality and reported as therapeutic targets for treatment of COVID-19 was significantly changed between controls and COVID-19 patients in the ICU (**Supplementary Figure 1B**) (47–49).

To examine differences in inflammatory pathways between COVID-19 patients and control ICU patients, we carried out Gene Set Variation Analysis (GSVA) (45) using a set of immune cell and pathway gene signatures (**Figure 1B**). Gene expression from COVID-19 patients was enriched for signatures of granulocytes, including inflammatory neutrophils and low-density granulocytes (LDGs) as well as plasma cells (PCs) and CD40 activated B cells. In addition, as compared to controls, COVID-19 patients exhibited decreased enrichment for signatures of dendritic cells (DCs), activated T cells, and Tregs.

### Enrichment of inflammatory cell types and pathway gene signatures separates COVID-19 ICU patients into two groups

**Figure 2:**
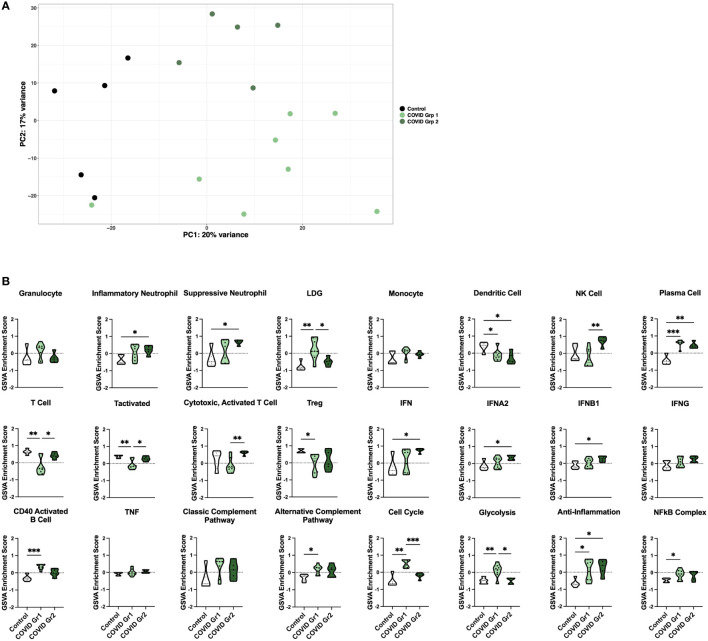
Enrichment of inflammatory cell types and pathway gene signatures in gene expression-derived COVID-19 AHRF patient groups. **(A)** Principle component analysis of the top 500 variable genes between control (black) and COVID-19 (green) ICU patients. COVID-19 patients were further separated into COVID Group 1 (light green) and COVID Group 2 (dark green). **(B)** Individual sample gene expression from **(A)** was analyzed by GSVA for enrichment of immune cell and pathway gene signatures. Enrichment scores are shown as violin plots. *p < 0.05, **p < 0.01, ***p < 0.001.

Principal component analysis (PCA) using the log_2_ transformed gene expression values differentiating COVID-19 and control ICU patients indicated a division among COVID-19 patients into two groups (**Figure 2A**). Notably, the two COVID-19 groups differed in expression of specific COVID-19 associated genes (**Supplementary Figure 1C**). COVID Group 1 patients tended to show an increase in the innate immune checkpoint molecule CD24, whereas COVID Group 2 patients had increased expression of the anti-viral response genes OAS1, OAS2, and OAS3.

We then utilized GSVA to examine inflammatory pathways in the two gene expression-derived COVID-19 patient groups in greater detail (**Figure 2B**). Enrichment of PCs and de-enrichment of DCs was conserved between both COVID-19 groups compared to controls. However, the majority of signatures were differentially enriched in the two groups, revealing distinct immune profiles. Specific granulocyte population signatures were enriched in the COVID-19 patient groups with increased LDGs in COVID Group 1 and increased inflammatory and suppressive neutrophils in COVID Group 2. In addition, COVID Group 1 was uniquely enriched for signatures of CD40 activated B cells, the alternative complement pathway, the cell cycle, glycolysis, and the NFkB complex and de-enriched for activated T cell signatures. In COVID Group 2, natural killer (NK) cell, general interferon (IFN), IFNA2, and IFNB1, but not IFNG signatures were significantly increased, whereas no inflammatory signatures were decreased compared to controls.

### Conserved and unique immune signatures identify ICU patients with different causes of AHRF

**Figure 3:**
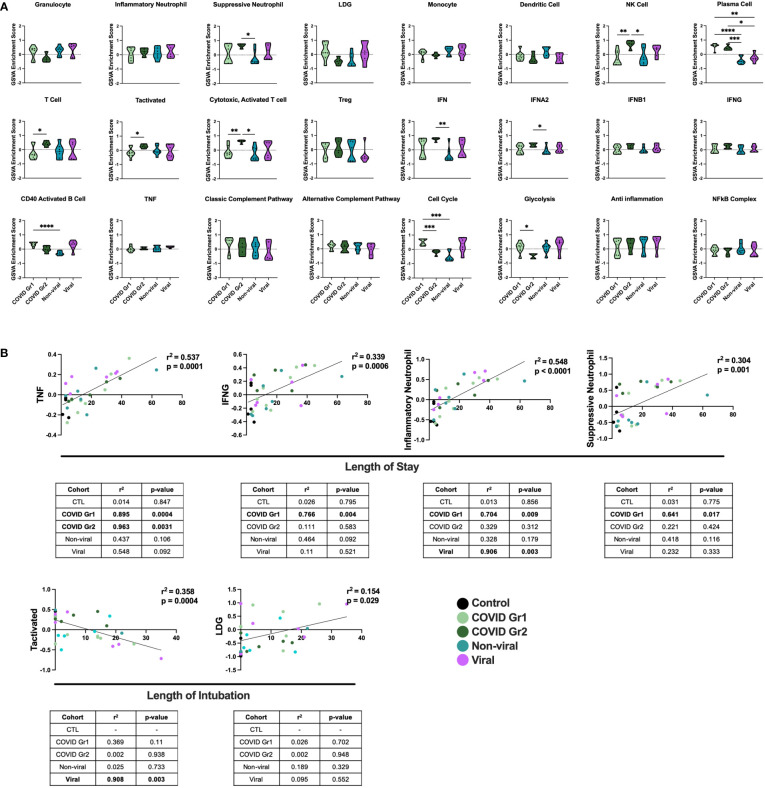
Conserved and unique immune signatures identify ICU patients with different sources of AHRF and vary in correlations with clinical data. **(A)** Individual sample gene expression from COVID Group 1, COVID Group 2, Viral, or Non-viral AHRF ICU patient cohorts was analyzed by GSVA for enrichment of immune cell and pathway gene signatures. Enrichment scores are shown as violin plots. *p < 0.05, **p < 0.01, ***p < 0.001, ****p < 0.0001. **(B)** Multivariable linear regression analysis of immune cell gene signatures significantly correlated with clinical data from Control, COVID Group 1, COVID Group 2, Viral, and Non-viral AHRF ICU patient cohorts. Combined cohort correlations and p-values are displayed in the linear regression plots while individual cohort correlations and p-values are displayed in the tables below. Correlations with p < 0.05 were considered significant.

We also compared gene signature enrichment from ICU patients with COVID-19-induced AHRF to patients admitted to the ICU with respiratory failure from other non-SARS-CoV-2 viral infections or non-viral causes (**Figure 3A**). The PC gene signature was consistently enriched in both COVID Group 1 and 2 compared with the non-viral and viral AHRF cohorts. Numerous differences in gene set enrichment patterns were noted between the COVID-19 groups and those with other causes of AHRF. Many of the differences in immune cell and pathway enrichment between COVID Group 1 and 2 and non-viral AHRF patients were consistent with the differences from control ICU patients, whereas, in general, viral and COVID-19 AHRF were more similar. In COVID Group 1, CD40 activated B cells and the cell cycle were increased over the non-viral AHRF group. In COVID Group 2, suppressive neutrophils, NK cells, T cells, IFN, IFNA2, and IFNB1 were increased, whereas granulocytes and glycolysis were decreased. as compared to non-viral AHRF. The most consistent difference between COVID Group 1 or COVID Group 2 and viral AHRF patients was the increased PC signature in the COVID patients.

To correlate GSVA gene signature enrichment with AHRF patient cohort and clinical features, we performed multivariable linear regression analysis using MaAsLin 2 and plotted relationships with significant correlations (46) (**Figure 3B**; **Supplementary Table 2**). As a result, we found the TNF, IFNG, inflammatory neutrophil, and suppressive neutrophil signatures had significant positive correlations with length of stay. In addition, the LDG signature had a significant positive correlation and the activated T cell signature a significant negative correlation with length of intubation. However, when linear regression analysis was carried out individually for each cohort, we found that the gene signature to clinical feature correlations were not significant for all patient cohorts (**Figure 3B**). None of these gene signature to clinical feature correlations were significant when considering the control ICU patients or non-viral AHRF patients alone, indicating that these relationships were specific for patients with virus-induced respiratory failure. Among viral and COVID AHRF cohorts, the positive correlation between inflammatory neutrophils and length of stay was significant for both COVID and viral AHRF patients while the correlations between TNF, IFNG, or suppressive neutrophils and length of stay were only significant for COVID but not viral AHRF cohorts. Furthermore, the negative correlation between activated T cells and length of intubation was only significant for the viral AHRF cohort, but not for the COVID groups. Therefore, subsets of ICU patients exhibit differences in immune signatures indicative of a worse clinical prognosis, but this is not unique for COVID-19 patients.

### Specific plasma cell populations are characteristic of COVID-19-induced AHRF

**Figure 4:**
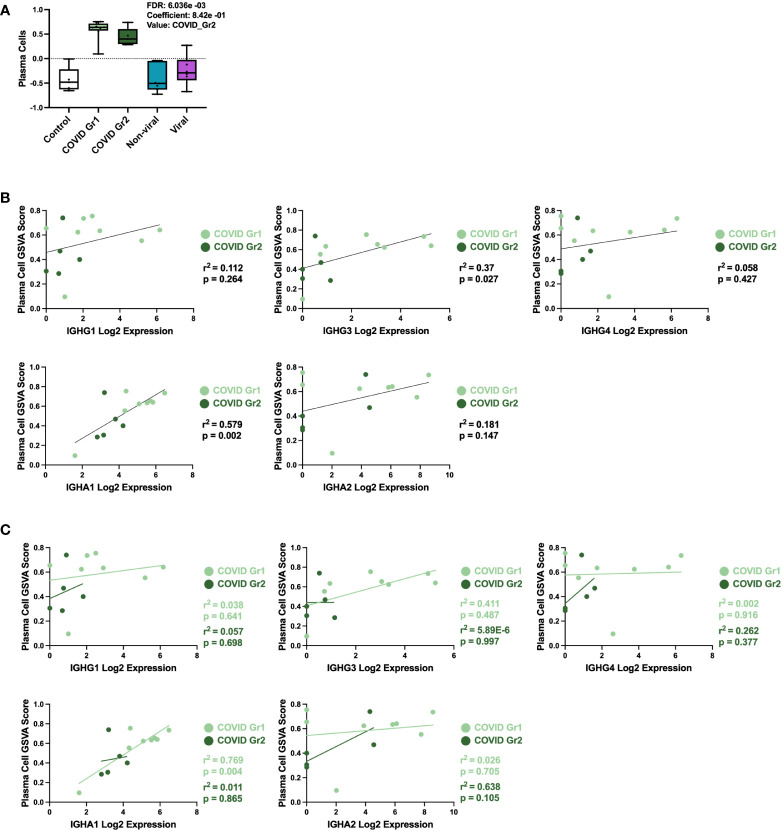
Specific plasma cell populations are characteristic of COVID-19-induced AHRF. **(A)** Multivariable linear regression analysis boxplots depicting significant correlation of the PC gene signature GSVA scores with ICU patient cohort. **(B, C)** Linear regression between PC GSVA scores and Ig heavy chain isotype log_2_ gene expression values for COVID Group 1 and COVID Group 2 ICU patient cohorts. Combined cohort correlations and p-values are depicted in **(B)** and individual cohort correlations and p-values are depicted in **(C)**. Correlations with p < 0.05 were considered significant.

COVID-19 patients, whether Group 1 or Group 2, had a significant increase in PCs over all other ICU patients. This result was further probed by multivariable linear regression analysis, in which the most significant correlation between GSVA enrichment score and patient cohort was for the PC signature, which was uniquely associated with COVID-19 patient groups (**Figure 4A**). To investigate the immunoglobulin (Ig) heavy chain(s) expressed by AHRF COVID-19 patient PCs, we carried out linear regression using PC GSVA scores and Ig heavy chain gene expression (**Figures 4B, C**). As a whole, COVID-19 patient PC GSVA scores were significantly correlated with IGHG3 and IGHA1 Ig heavy chain isotypes (**Figure 4B**). However, when the COVID groups were analyzed separately, only COVID Group 1 showed a significant correlation with expression of IgHA1 (r^2 =^ 0.8, p=0.004). This was not found with COVID group 2 (**Figure 4C**).

### Clinical features and serum cytokines are indicative of differential disease severity in gene expression-derived COVID-19 patient groups

**Figure 5:**
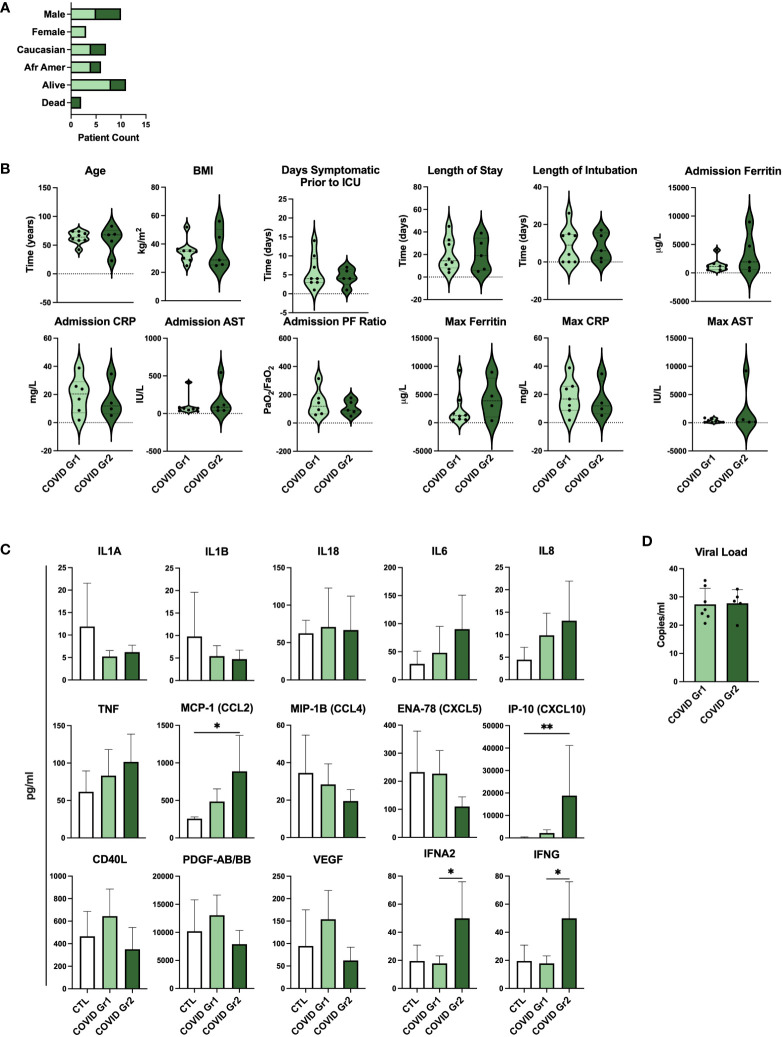
Serum cytokines, but not viral load, are indicative of differential disease severity in gene expression-derived COVID-19 patient groups. **(A)** Demographic data and **(B)** clinical feature data from COVID Group and COVID Group 2 patient cohorts. **(C)** Serum cytokine measurements from Control, COVID Group 1, and COVID Group 2 ICU patient cohorts. **(D)** SARS-CoV-2 viral load CT values of nasal swabs from COVID-19 ICU patient cohorts. *p < 0.05, **p < 0.01.

To determine whether gene expression-derived groups of hospitalized COVID-19 patients also differed in the level of disease severity, we compared clinical feature and cytokine data of COVID Group 1 and Group 2 patients (**Figure 5**; **Supplementary Table 3**). We found that baseline demographic data as well as length of stay in ICU and length of intubation were similar between COVID Group 1 and 2 (**Figures 5A, B**). Notably, however, these cohorts varied widely in a number of clinical features indicating that COVID Group 2 had more severe disease (**Figure 5B**). On average, COVID Group 2 had two fewer days of symptoms before admission to the ICU and thus had accelerated disease onset. Upon admission, ferritin and AST levels were over 2X and 1.5X higher, respectively, in Group 2 patients whereas their lung function, as measured by mean PF ratio, was lower. Furthermore, maximum ferritin and aspartate aminotransferase (AST) levels were even more elevated in COVID Group 2 than at admission, indicative of rapid disease progression in these patients. In contrast to clinical features, pro-inflammatory cytokines were only modestly elevated in COVID Group 1 and 2 over controls and in COVID Group 2 over Group 1 (**Figure 5C**). COVID Group 1 and 2 exhibited modest increases in IL6, IL8, and TNF, although these differences did not reach statistical significance. In addition, COVID Group 1 had slightly elevated CD40L and VEGF and COVID Group 2 had significantly elevated levels of the myeloid chemokines CCL2 and CXCL10 as well as IFNA2 and IFNG. In many cases, severe COVID-19 patients are thought to have had greater viral exposure and thus greater viral load in relation to mild cases (50, 51). However, we found no significant differences in viral loads between the COVID-19 patient groups despite their clear differences in clinical features and gene expression profiles (**Figure 5D**).

### Longitudinal sampling reveals persistence of immune cell and pathway gene signatures in AHRF ICU patients over time

**Figure 6:**
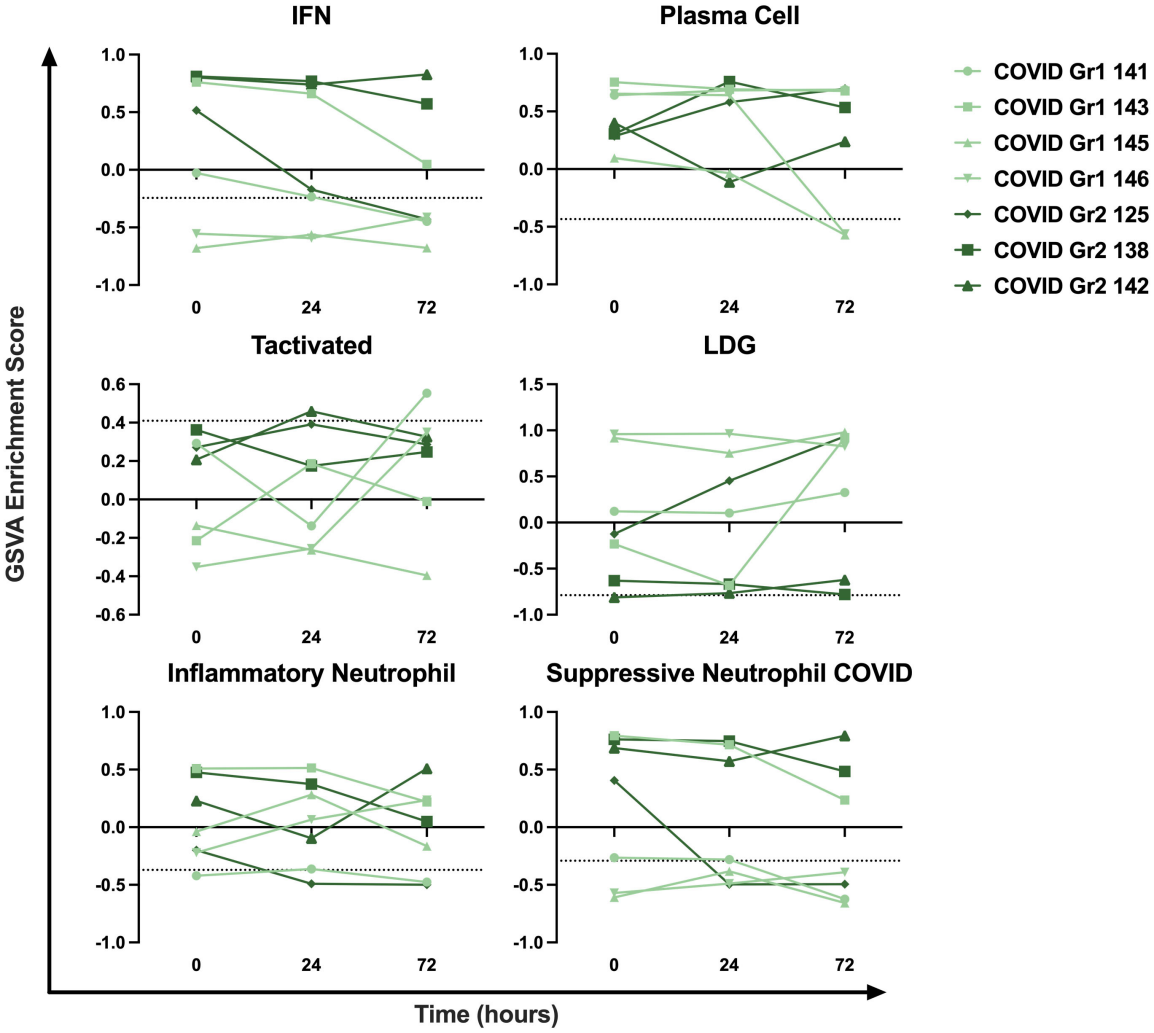
Longitudinal sampling reveals persistence of immune cell and pathway gene signatures over time. Trajectory plots of select immune cell and pathway GSVA enrichment scores from individual COVID-19 ICU patients at baseline, 24 hours, and 72 hours post-admission.

To examine the persistence of gene signature enrichment over time in the ICU, several COVID-19 AHRF, Viral AHRF, and Non-viral AHRF patients were also sampled at 24- and 72-hours post-admission and individualized trajectories of gene expression were assessed (**Figure 6** and **Supplementary Figure 2**). Gene signatures for all AHRF cohorts remained largely stable over time, but among individuals with COVID-19, Group 1 patients appeared to have greater variation than Group 2 patients. In particular, the IFN and neutrophil signatures that were uniquely enriched in COVID Group 2 were decreased in Group 1 patients over time, whereas the LDG signature was increased. Importantly, Group 2 patient 142, who succumbed, displayed very little change in gene expression over the 72 hours.

### Non-hospitalized COVID-19 patient gene expression profiles resemble healthy controls, particularly at later stages of disease

**Figure 7:**
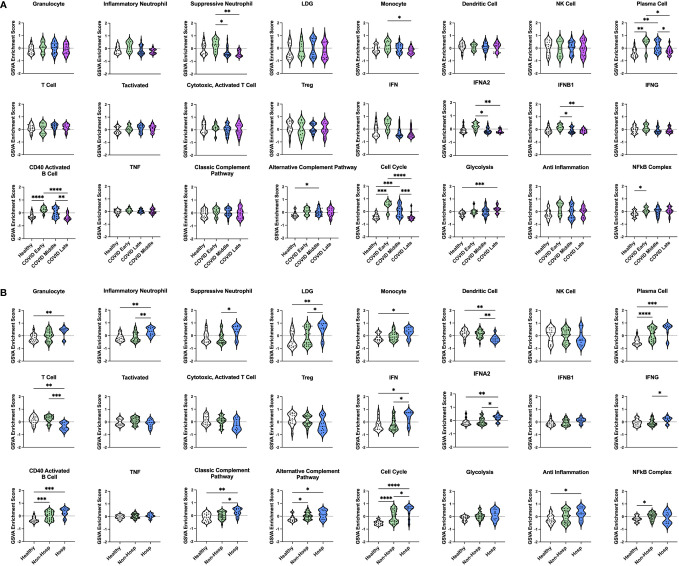
Enrichment of immune cell and pathway gene signatures in non-hospitalized and hospitalized COVID-19 patients at different stages of disease. **(A)** Individual sample gene expression from non-hospitalized COVID-19 patients with early-, mid-, or late-stage disease and healthy controls was analyzed by GSVA for enrichment of immune cell and pathway gene signatures. **(B)** Individual sample gene expression from non-hospitalized and hospitalized COVID-19 patients and healthy controls was analyzed by GSVA for enrichment of immune cell and pathway gene signatures. Enrichment scores are shown as violin plots. *p < 0.05, **p < 0.01, ***p < 0.001, ****p < 0.0001.

Our initial dataset of COVID-19 patients consisted entirely of severe AHRF cases admitted to the ICU. Therefore, we wanted to characterize the immune profiles of COVID-19 patients at different stages of diseases and severity (non-hospitalized vs hospitalized) as compared to healthy controls. To do this, we analyzed a second publicly available COVID-19 transcriptomic dataset (GSE161731, **Supplementary Table 1**), which sampled 16 COVID-19 patients at early-stage (< 10 days), 30 at mid-stage (11-21 days), and 17 at late-stage (> 21 days) disease (52). In total, there were 82 common DEGs between individuals with COVID-19 and healthy controls in this study and our analysis of COVID-19 ICU patients compared to controls in the ICU (**Supplementary Figure 3**). GSVA analysis revealed that many gene signatures enriched in AHRF COVID-19 patients were selectively enriched in the early and mid-stage, but not late-stage disease cohorts (**Figure 7A**). Furthermore, early-stage patients most resembled the COVID Group 2 cohort, whereas mid-stage disease patients resembled COVID Group 1. Early stage COVID-19 patients were enriched for suppressive neutrophil, monocyte, PC, IFN, CD40 activated B cell, cell cycle, and NFkB gene signatures. Mid-stage patients were enriched for PC, CD40 activated B cell, alternative complement pathway, and cell cycle gene signatures. Late-stage patients were de-enriched for all of these signatures as compared to the early and mid-stage disease cohorts and had no significant differences from healthy controls. Notably, while early and mid-stage cohorts included both mild, non-hospitalized and severe, hospitalized patients, none of the patients with late-stage disease required hospitalization.

### Immune cell and pathway gene signature enrichments are conserved between hospitalized COVID-19 patients

In addition to differences in disease stage, patients with early and mid-stage disease were further differentiated by disease severity based on whether they were hospitalized. Comparing 35 non-hospitalized and 11 hospitalized COVID-19 patients (**Figure 7B**) revealed that a few signatures were commonly enriched in all COVID-19 patients regardless of disease severity, including PCs, CD40 activated B cells, the alternative complement pathway, and the cell cycle. Interestingly, linear regression analysis of PC GSVA scores with IgH chain gene expression revealed that both non-hospitalized and hospitalized COVID-19 patients had significant correlations with IgG and IgA chain genes, although the non-hospitalized patient correlations were stronger than those of hospitalized patients (**Supplementary Figure 4**). The NFkB complex signature was the only one uniquely enriched in non-hospitalized COVID-19 patients as compared to healthy controls. In contrast, many inflammatory signatures were specific to hospitalized COVID-19 patients, including increased granulocyte, inflammatory neutrophil, suppressive neutrophil, LDG, monocyte, IFN, classic complement pathway, and anti-inflammation signatures. In addition, only hospitalized patients had decreased DC and T cell gene signatures. To provide further support for these results, we analyzed a third publicly available dataset (GSE172114) of 23 non-critical and 46 critical COVID-19 patients. Critical COVID-19 patients from this study shared 103 and 2573 DEGs with our previous analyses of COVID-19 ICU patients and COVID-19 patients with varying disease severity respectively (**Supplementary Figure 3**). Notably, the enriched gene signatures previously noted in hospitalized compared to non-hospitalized COVID-19 patients were also enriched in this group of critical compared with non-critical COVID-19 patients (**Supplementary Figure 5**). Therefore, severe cases of COVID-19, which require hospitalization, have conserved immune profiles as measured by inflammatory gene signatures, but upon further dissection reveal patient heterogeneity indicative of risk for more severe disease.

## Discussion

Bioinformatic analysis of gene expression data from COVID-19 patients of varying disease stage and severity was used to identify immune signatures common to COVID-19 as well as immune signatures that differentiate patients with severe disease requiring hospitalization. Shared enrichment of the PC gene signature was the one constant feature of COVID-19 patients in comparison to healthy controls, regardless of the length of time since symptom onset or whether their disease was severe enough to require hospitalization, which is consistent with previous work from our group and others (3, 19, 36, 53). This rapid increase in the PC signature suggests that SARS-CoV-2 infection initiates a robust generation of antibody secreting cells (ASCs), potentially through an extrafollicular response, which are capable of producing virus-specific neutralizing antibodies (54, 55). These ASCs are presumably generated in secondary immune organs and migrate to the bone marrow as a normal feature of immunization. However, as fully mature PCs are rare to find in the blood, this enrichment likely represents an increase in short-lived plasmablasts (PBs), which are still capable of producing neutralizing antibodies, but may not have undergone somatic hypermutation to increase their specificity for SARS-CoV-2 viral antigens (56). Thus, the increased PC signature may be largely COVID, but not severity -specific and could account for conflicting reports as to whether antibody production in COVID-19 is helpful or harmful (53).

The pathogenic gene signatures associated with hospitalized COVID-19 patients were conserved across multiple datasets, suggesting that these enrichments represent a common immune profile of severe COVID-19 (52). This profile was characterized by increased enrichment of neutrophil subsets and IFN accompanied by de-enrichment of T cells and was specific to patients with early and mid-stage disease (< 21 days since symptom onset). Notably, the greatest inflammatory signature enrichment was observed in early-stage patients (< 10 days since symptom onset). In contrast, the immune profiles of patients who reached late-stage disease (> 21 days since symptom onset) were no different than healthy controls and none of these individuals were hospitalized indicating that they would fully recover. Thus, this result stresses the importance of early identification of infected individuals and provides critical insight into the pathologic immune signatures that are risk factors for the development of severe disease and need for hospitalization.

Analysis of longitudinal gene expression data from patients admitted to the ICU was utilized to decode the heterogeneity among severe COVID-19 patients and to differentiate them from others with AHRF based on their immune profiles. As a result, we identified and characterized 2 groups of COVID-19 patients (COVID Group 1 and COVID Group 2), with conserved and differential enrichment of immune cell and pathway gene signatures. COVID Group 1 was characterized by a lack of activated T cells, increased LDGs, increased CD40-activated B cells, and a general increase in cell proliferation and metabolism pathways. COVID Group 2 was characterized by increased expression of neutrophil subsets, markedly increased IFN gene signatures, and the absence of IgA1 expressing PCs. Aggregated clinical feature data and cytokine profiles for each COVID-19 patient cohort revealed that COVID Group 2 appeared to have more severe disease outcomes and indicated that patients with a similar immune profile would warrant a more targeted and aggressive therapeutic approach to mitigate risk of mortality.

Both COVID Group 1 and 2 shared enrichment of the PC signature, which consistently differentiated COVID-19 patients from control ICU patients as well as patients exhibiting respiratory failure from other viral or non-viral sources. However, it is possible that the IgH heavy chain isotype of PCs is indicative of whether a robust and early PC expansion is beneficial to COVID-19 patients and certain PC isotypes have been linked with severe disease (19). In line with this, we found evidence of differing PC specificities in COVID Group 1 and 2 patients as Group 2 patients, who exhibited more severe clinical features, also appeared to fail to generate an IgA1 PC response. This suggests that Group 2 patients may have a defect in T-B cell collaboration and the ability to produce class-switched IgA1 PCs. The IgA response is important to clear virus from mucosal surfaces, such as the lung and, therefore, a lack of IgA in COVID Group 2 may compromise SARS-CoV-2 clearance in these patients (57). Furthermore, production of autoantibodies of varying specificities has been reported in COVID-19 patients and could represent an non-specific PC response that contributes to systemic inflammation in infected individuals (33, 58). Therefore, a better characterization of the nature of PC generation and function following SARS-CoV-2 infection could be a critical factor in understanding the host immune response and how it differs among individuals.

COVID Group1 patients appeared to have less severe disease as compared to COVID Group 2. Whereas all presented with AHRF, all Group 1 patients recovered, whereas 2 of Group 2 patients died during their hospitalization. Although our data set is limited by the number of patients analyzed, it suggests that the Group 2 gene signature could serve as prognostic marker and warrant individualized intervention. Lymphopenia is an established feature of COVID-19 and, in particular, a lack of T cell responses has been associated with worse clinical outcome (14, 28, 53, 59, 60). However, COVID Group 1 patients had differential enrichment of B and T cell populations with enrichment of CD40 activated B cells and de-enrichment of activated and cytotoxic T cells. In addition, unlike non-viral and viral AHRF patient cohorts, lack of activated T cells failed to correlate with clinical data. This would indicate that a lack of T cell activation and function is detrimental to patient outcome, but not essential for patient recovery and also that a robust activated B cell response may be able to compensate in some capacity.

COVID Group 1 patients also exhibited an increase in genes associated with LDGs, neutrophil-like granulocytes with enhanced capacity for production of Type I IFNs and formation of neutrophil extracellular traps (NETs) that have been identified in severe COVID-19 patients (61, 62). In agreement with reports that NET formation contributes to enhanced pathogenesis in COVID-19 patients, it is likely that enrichment of LDGs contributes to the development of AHRF (63, 64). However, the lack of LDG enrichment in COVID Group 2 patients suggests that enrichment of LDGs does not increase the risk of death. In fact, the absence of an extreme IFN response and eventual recovery of all COVID Group 1 patients, suggests that the increased LDG signature reflects an appropriate antiviral innate immune response that will eventually subside as the virus is cleared. This was supported by the elevated expression of CD24 observed in COVID Group 1 patients, as the CD24-SIGLEC10 signaling axis serves as an important regulator of innate immune. Notably, CD24Fc was effective in protection against viral pneumonia in a simian model and has been proposed for the treatment of COVID-19 (49).

In contrast to COVID Group 1, the immune response of COVID Group 2 patients appeared to be associated with increased risk of mortality. The primary immune signatures enriched in COVID Group 2 resembled a dysregulated antiviral innate immune response. In particular, Group 2 exhibited enrichment of neutrophil populations expressing pro-inflammatory and suppressive genes that were previously identified in blood from severe COVID-19 patients (13, 18). Furthermore, levels of cytokines and chemokines with roles in myeloid cell activation and recruitment were significantly elevated and could contribute to aberrant expansion of these pathogenic neutrophils and disease progression. COVID Group 2 patients also had significant enrichment of Type I IFN gene signatures and increased serum levels of IFN proteins compared to COVID Group 1. To date, there have been conflicting reports claiming that severe COVID-19 cases exhibit increased (29) or impaired (27) Type I IFN responses. However, our results would suggest that severe COVID-19 patients exhibit a range of IFN responses, but that extreme early IFN production ultimately increases risk of death.

In addition to IFNs, a number of pro-inflammatory cytokines, including members of the IL-1 family, IL-6, IL-8, and TNF have been implicated in COVID-19 pathogenesis and linked to severe disease (14, 24, 35). We also observed that severe COVID-19 patients in the ICU had a trend toward increases in IL-6, IL-8, and TNF over control ICU patients and this increase was even greater in COVID Group 2 over Group 1. However, there was considerable heterogeneity and none of these comparisons reached statistical significance. This result corroborates a number of reports that have questioned the notion that “cytokine storm” is a prominent contributor to COVID-19 pathogenesis (17, 21). Viral load has also had conflicting associations with disease severity (50, 51). However, we found no difference in viral load between our COVID-19 patient cohorts suggesting that greater mortality risk is not necessarily associated with greater viral exposure. One caveat to this is that our viral load measurements were taken from nasal swabs and it is possible that increased viral presence in the lower airway may lead to worse disease outcomes. The lack of a clear association between more severe clinical manifestations and accentuated gene expression profiles and viral load suggests that genetic control of host defense may play a prominent role in disease outcome, as has been suggested (47, 48, 65).

Longitudinal gene expression analysis over 72 hours after admission to the ICU revealed that the immune profiles of COVID and non-COVID AHRF patients remained largely unchanged over time. However, among the COVID-19-induced AHRF patients, profiles of COVID Group 1 patients appeared to exhibit greater changes than COVID Group 2. Strikingly, gene signatures of COVID-associated neutrophil subsets and IFN were decreased, whereas the LDG gene signature was increased in COVID Group 1 patients, further supporting the conclusion that the innate immune response in Group 1 patients contributes to viral clearance whereas the response in Group 2 patients contributes to enhanced inflammation and fatal disease.

We have applied a combination of bioinformatics approaches to characterize COVID-19 patients based on disease stage and severity using gene expression data, but must acknowledge the limitations of the data. Our initial dataset of ICU patients contained up to 13 patients per cohort and COVID-19 patients were also sub-divided into groups, which reduced the statistical power of our analyses. To mitigate this, we designed the study to solely include patients in the ICU, including non-AHRF controls, and thus reduce patient heterogeneity. In addition, a larger publicly available dataset including both hospitalized and non-hospitalized COVID-19 patients with healthy controls was utilized for validation. However, additional studies comparing ICU patients with more patients per cohort and more uniform inclusion criteria are warranted.

Overall, we have identified immune profiles of severe COVID-19 patients associated with full recovery from disease or increased risk of mortality. We propose that these differences in COVID Group 1 and Group 2 patients could be employed to better allocate healthcare resources and design targeted treatment plans to better care for individuals who are at the greatest risk of worse outcomes. Whereas optimal medical care and appropriate ventilator management may be sufficient in patients with immune profiles similar to COVID Group 1, patients with immune profiles similar to COVID Group 2 could benefit from more aggressive therapeutic intervention targeting the dysregulated innate immune response. In particular, drugs targeting Type I IFNs, cytokines, such as IL6 or TNF, or myeloid chemokines such as IP-10 or MCP1 could be effective treatments for these individuals. Our work highlights the heterogeneity among severe cases of COVID-19 and the need for better characterization of hospitalized individuals to determine effective strategies to mitigate pathogenic immune processes that are dysregulated in the most at-risk patients. Furthermore, infected individuals with the potential to progress to severe disease should be identified as early as possible to allow for better resource allocation and early individualized therapies.

## Data availability statement

Data deposited in NCBI under BioProject accession PRJNA777938.

## Ethics statement

The studies involving human participants were reviewed and approved by UVA Institutional Review Board for Health Sciences Research. The patients/participants provided their written informed consent to participate in this study.

## Author contributions

Conceptualization: CB, LS, JS, AG, AK, and PL. Methodology: CB, LS, JS, AK, and PL. Software: PB. Formal Analysis: AD and PB. Data Curation: PB, CB, LS, JS, and AK. Writing – Original Draft: AD. Writing – Review & Editing, AD, CB, LS, JS, AK, and PL. Visualization: AD. Supervision: AD, AG, AK, and PL. Project Administration: AG, AK, and PL. Funding Acquisition: AK and AG. All authors contributed to the article and approved the submitted version.

## Funding

NIH NHLBI K23 HL143135 (CB). NIH NIAID 1R21AI160334 (CB). COVID-19 Rapid Response Initiative by the Global Infectious Disease Institute, University of Virginia (CB, LS, AK). The RILITE Foundation (AG, PL).

## Acknowledgments

We thank the patients who provided samples for this study as well as the authors of previous studies who made their data publicly available and facilitated our analyses.

## Conflict of interest

Authors AD, PB, AG and PL were employed by company AMPEL BioSolutions LLC.

The remaining authors declare that the research was conducted in the absence of any commercial or financial relationships that could be construed as a potential conflict of interest.

## Publisher’s note

All claims expressed in this article are solely those of the authors and do not necessarily represent those of their affiliated organizations, or those of the publisher, the editors and the reviewers. Any product that may be evaluated in this article, or claim that may be made by its manufacturer, is not guaranteed or endorsed by the publisher.
