# Supplementary material for: COVID-19 patients exhibit unique transcriptional signatures indicative of disease severity
Source: Front Immunol. 2022 Sep 15;13:989556. doi: 10.3389/fimmu.2022.989556 (PMC9522616; doi:10.3389/fimmu.2022.989556)
Supplement: Supplementary file 1 [file DataSheet_1.pdf]

Supplementary Figures

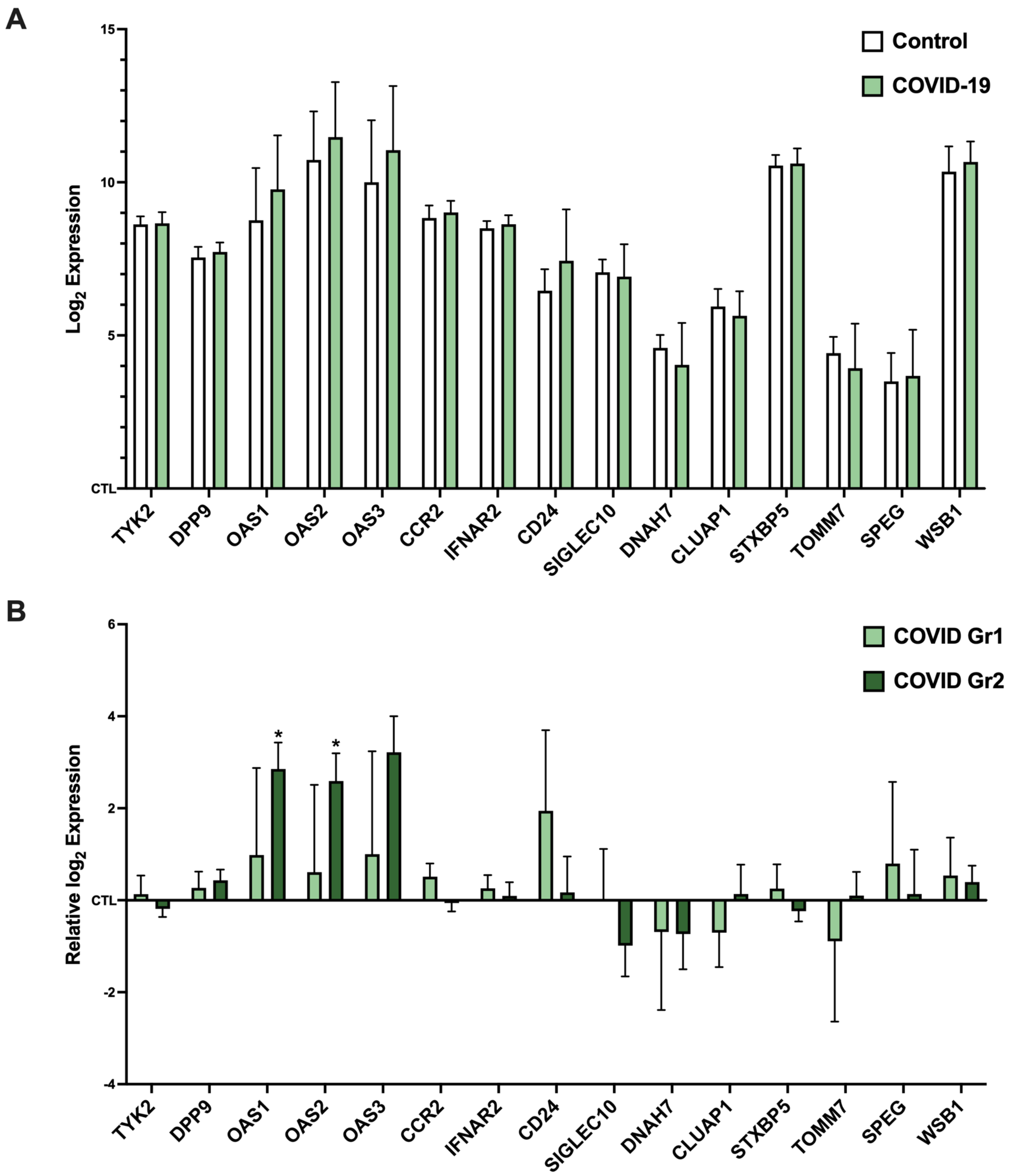

Supplementary Figure 1. Expression of genes associated with disease severity and mortality in AHRF COVID-19 patients. A) RNA-seq log_2 expression values for genes identified in previous studies as indicators of COVID-19 disease severity or mortality [37-39] from control and COVID AHRF patients upon admission to the ICU. (B) Relative log_2 expression of genes in (A) from gene expression-derived COVID-19 patient groups normalized to expression in control ICU patients. $*p<0.05$

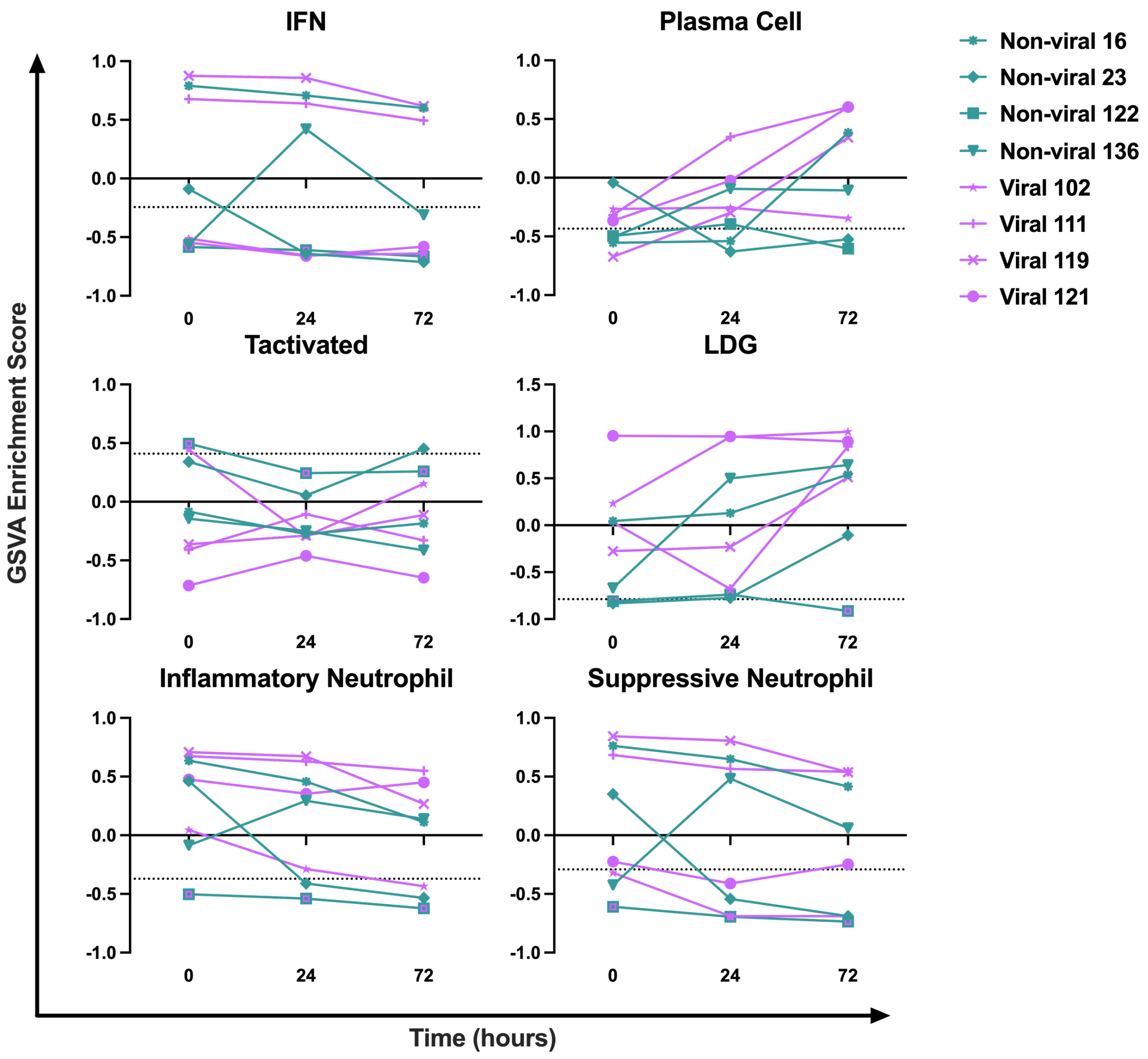

Supplementary Figure 2. Longitudinal sampling of viral and non-viral AHRF patients. Trajectory plots of select immune cell and pathway GSVA enrichment scores from individual Viral and Non-Viral AHRF ICU patients at baseline, 24 hours, and 72 hours post-admission.

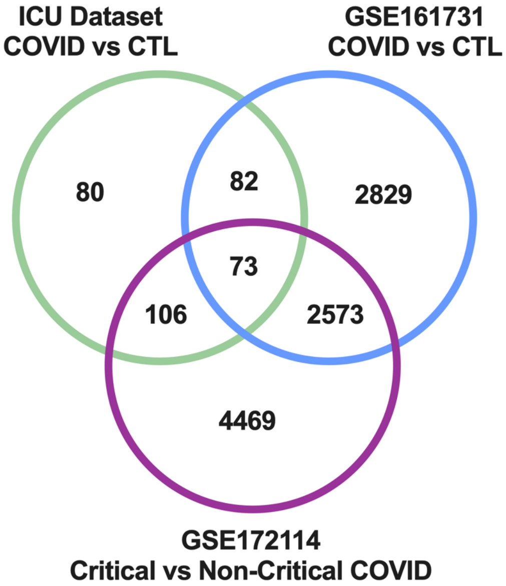

Supplementary Figure 3. Comparison of DEGs between COVID-19 patients and controls across multiple studies. Venn diagram of differentially expressed genes ($p<0.05$) between COVID-19 patients and controls from the indicated RNA-seq datasets.

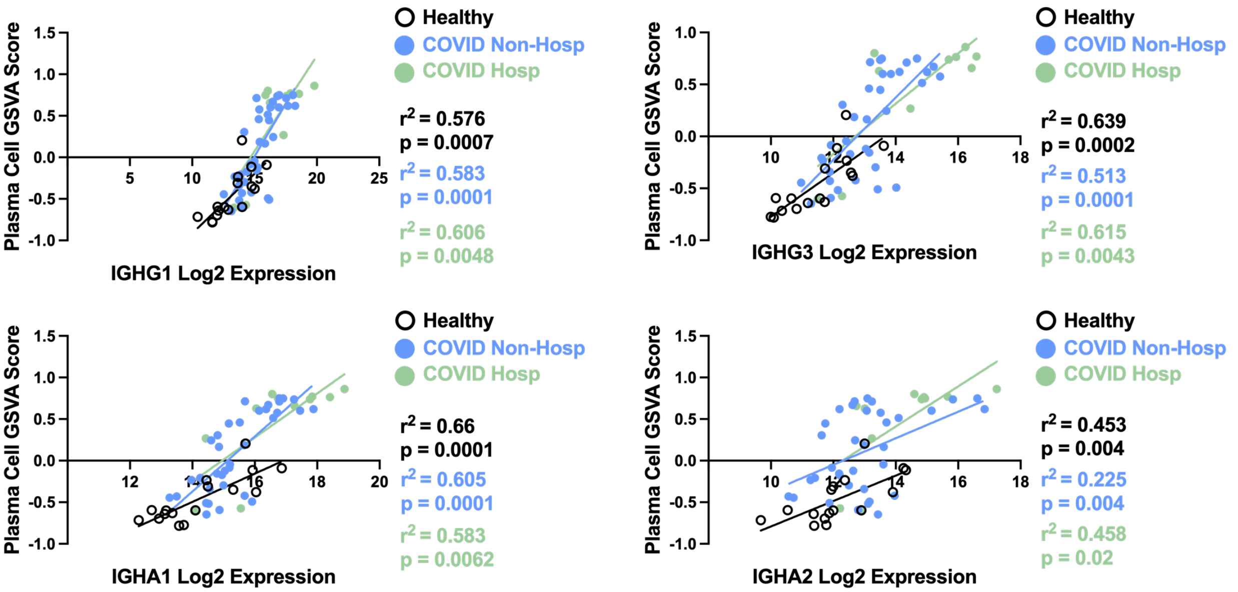

Supplementary Figure 4. Plasma cell isotype analysis of non-hospitalized and hospitalized COVID-19 patients. Linear regression between PC GSVA scores and IgH chain isotype log_2 gene expression values for non-hospitalized and hospitalized COVID-19 patients and healthy controls. Correlations and p-values are displayed for each individual cohort. Correlations with $p<0.05$ were considered significant.

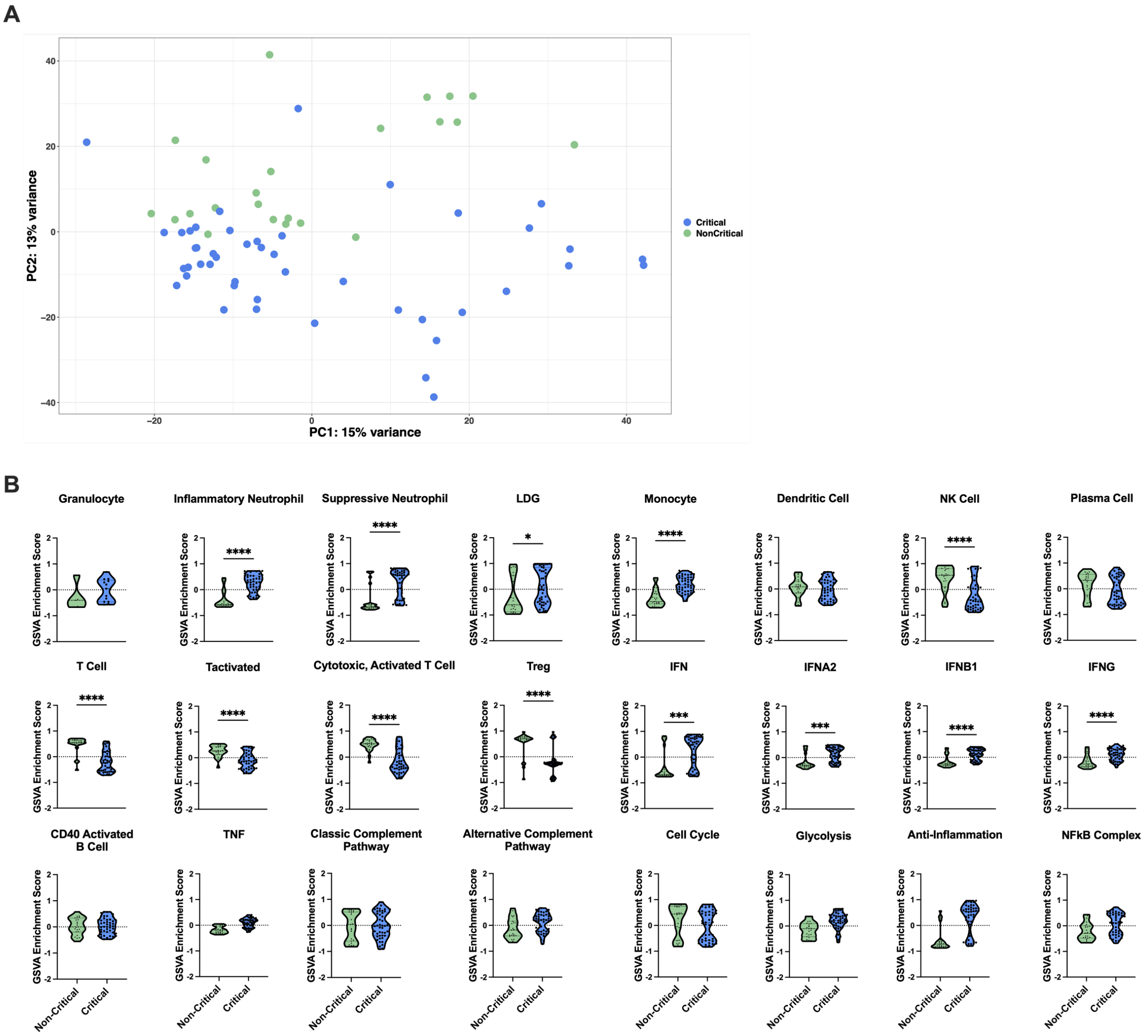

Supplementary Figure 5. Immune profiles of critical and non-critical COVID-19 patients. (**A**) Principle component analysis of the top 500 variable genes between critical (blue) and non-critical (green) COVID-19 patients. (**B**) Individual sample gene expression from (**A**) was analyzed by GSVA for enrichment of immune cell and pathway gene signatures. Enrichment scores are shown as violin plots. *$p<0.05$, **$p<0.01$, ***$p<0.001$
